# Optimizing multiplex SNP-based data analysis for genotyping of *Mycobacterium tuberculosis* isolates

**DOI:** 10.1186/1471-2164-15-572

**Published:** 2014-07-07

**Authors:** Sarah Sengstake, Nino Bablishvili, Anja Schuitema, Nino Bzekalava, Edgar Abadia, Jessica de Beer, Nona Tadumadze, Maka Akhalaia, Kiki Tuin, Nestani Tukvadze, Rusudan Aspindzelashvili, Elizabeta Bachiyska, Stefan Panaiotov, Christophe Sola, Dick van Soolingen, Paul Klatser, Richard Anthony, Indra Bergval

**Affiliations:** KIT Biomedical Research, Royal Tropical Institute, Meibergdreef 39, 1105 AZ Amsterdam, The Netherlands; National TB Reference Laboratory, National Center for Tuberculosis and Lung Diseases, 50 Maruashvili Street, 0101 Tbilisi, Georgia; Instituto Venezolano de Investigaciones Cientificas (IVIC), Laboratorio de Genética Molecular, CMBC, Caracas, Venezuela; Institute of Genetics and Microbiology, UMR 8621 CNRS/UPS11, Buildings 400 et 409, Faculty of Sciences - University Paris-Sud 11, 15, rue Georges Clémenceau, 91405 Orsay, France; Tuberculosis Reference Laboratory, Centre for Infectious Disease Control, Centre for Infectious Disease Research, Diagnostics and Perinatal Screening, National Institute for Public Health and the Environment, P.O. Box 1, 3720 BA Bilthoven, The Netherlands; National Reference Laboratory, National Center for Tuberculosis Problems, Ministry Health of the Republic Kazakhstan, 5, Bekhozhin str, Almaty, 050010 Republic of Kazakhstan; MRC-Holland, Willem Schoutenstraat 6, 1057 DN Amsterdam, The Netherlands; National Center of Infectious and Parasitic Diseases, 26 Yanko Sakazov blvd, Sofia, 1504 Bulgaria; Tuberculosis Reference Laboratory, Centre for Infectious Disease Control, National Institute for Public Health and the Environment, P.O. Box 1, 3720 BA Bilthoven, The Netherlands; Department of Pulmonary Disease, University Centre for Chronic Diseases, and Department of Medical Microbiology, Radboud University Nijmegen Medical Centre, P.O. Box 9101, 6500 HB Nijmegen, Netherlands

**Keywords:** *Mycobacterium tuberculosis*, MLPA, Data analysis, SNP typing, MAGPIX, Drug resistance, MTBC lineage, Republic of Georgia

## Abstract

**Background:**

Multiplex ligation-dependent probe amplification (MLPA) is a powerful tool to identify genomic polymorphisms. We have previously developed a single nucleotide polymorphism (SNP) and large sequence polymorphisms (LSP)-based MLPA assay using a read out on a liquid bead array to screen for 47 genetic markers in the *Mycobacterium tuberculosis* genome. In our assay we obtain information regarding the *Mycobacterium tuberculosis* lineage and drug resistance simultaneously. Previously we called the presence or absence of a genotypic marker based on a threshold signal level. Here we present a more elaborate data analysis method to standardize and streamline the interpretation of data generated by MLPA. The new data analysis method also identifies intermediate signals in addition to classification of signals as positive and negative. Intermediate calls can be informative with respect to identifying the simultaneous presence of sensitive and resistant alleles or infection with multiple different *Mycobacterium tuberculosis* strains.

**Results:**

To validate our analysis method 100 DNA isolates of *Mycobacterium tuberculosis* extracted from cultured patient material collected at the National TB Reference Laboratory of the National Center for Tuberculosis and Lung Diseases in Tbilisi, Republic of Georgia were tested by MLPA. The data generated were interpreted blindly and then compared to results obtained by reference methods. MLPA profiles containing intermediate calls are flagged for expert review whereas the majority of profiles, not containing intermediate calls, were called automatically. No intermediate signals were identified in 74/100 isolates and in the remaining 26 isolates at least one genetic marker produced an intermediate signal.

**Conclusion:**

Based on excellent agreement with the reference methods we conclude that the new data analysis method performed well. The streamlined data processing and standardized data interpretation allows the comparison of the *Mycobacterium tuberculosis* MLPA results between different experiments. All together this will facilitate the implementation of the MLPA assay in different settings.

**Electronic supplementary material:**

The online version of this article (doi:10.1186/1471-2164-15-572) contains supplementary material, which is available to authorized users.

## Background

Bacterial genotyping has a recognized potential to support tuberculosis (TB) control [1]. After initial diagnosis and before administered standardized empirical therapy timely detection of resistance mutations in the genome of *Mycobacterium tuberculosis* (MTB) can help clinical decision-making and support infection control efforts. Further, combined resistance and lineage identification is especially interesting in areas where there is a high prevalence of multidrug resistant TB (MDR-TB) and resistance is associated with specific lineages [2–4]. The association between lineage type, patient outcome, and drug resistance needs to be studied to improve future control measures. Unfortunately the benefits of strain identification are seldom optimally realized as mycobacterial genotyping, especially lineage identification, is almost always performed retrospectively on sampled isolates in high burden settings.

MTB evolves unidirectionally by the accumulation of mutations that are then fixed with no evidence of genetic exchange [5, 6]. Drug resistance in MTB is almost exclusively conferred via SNPs [7]. Sets of SNPs and LSPs have also been identified as suitable markers for lineage identification within the MTB complex [8–10]. Consequently, SNP- and LSP-based assays are ideal for combined drug resistance testing and genotyping of MTB. We have previously developed and validated a bead-based multiplex ligation-dependent probe amplification (MLPA) assay and demonstrated its potential to simultaneously identify a range of drug resistance markers, discriminate within the genetic group MTB complex (MTBC), and detect and identify the clinically most relevant non-tuberculous mycobacterial species in cultured isolates [10–12].

MLPA assays or MLPA-like assays have been developed and reported by others for TB [20], other infectious agents [13–15], human DNA or RNA [16–18]. However, the bead-based tuberculosis-specific MLPA is unique in having a multiplexing capacity of up to 50 markers and combined detection of drug resistance and MTB lineage in a single assay. For high multiplexing assays a consistency check, i.e. the presence of sufficient markers and only markers exclusively from a single lineage, and streamlined data interpretation becomes increasingly useful and important as the numbers of markers screened increases. In previous reports we made use of a threshold value to classify fluorescent signals as positive or negative (see Figure four in, [10]). This threshold was based on preliminary testing of DNA from MTBC cultures and allowed accurate identification of the presence or absence of targeted markers in the majority of isolates tested [10]. However for signals that are close to the threshold automated interpretation of lineage types and drug resistance profiles can lead to a small proportion of unidentified false positives/false negatives which are not flagged as low confidence calls. As we perform a consistency check some of these calls will be identified, since many TB lineage markers are mutually exclusive, but for drug resistance markers this type of checking is not appropriate as mutations may occur in any lineage [19] and multiple mutations can be present in different combinations. To overcome this issue different approaches have been applied; for example adjusting the threshold value for all markers individually, making use of the signal-to-noise-ratio [15], reference genes [18], or calculating allelic ratios [20]. Here we have applied intra-sample normalization and inter-sample correction to allow low confidence calls to be identified and flagged for scrutiny. Identifying intermediate signals allows only the high confidence calls to be classified as positive and negative. Automated interpretation for profiles with only high confidence calls can then be trusted without the need to be reviewed by an expert.

The Republic of Georgia is reported as one of the 18 high-priority countries in the WHO European Region with the highest TB burden [21]. Additionally in 2011, 15.8% of all diagnosed pulmonary TB patients were reported with at least MDR-TB of which 6.2% were infected with an extensively resistant TB strain. We collected samples from patients diagnosed with pulmonary TB at the National TB Reference Laboratory (NRL) of the National Center for Tuberculosis and Lung Diseases (NCTBLD) in Tbilisi, Georgia. In addition to smear microscopy and chest X-ray, phenotypic drug susceptibility testing (DST) and molecular drug resistance testing by GenoTypeMTBDR*plus* assay [22–24] is performed routinely in this setting leading to the continuous reporting of MTB surveillance data. In contrast there are presently no typing methods established in this setting and epidemiological data regarding TB lineage type are only assessed retrospectively outside of the country [25–27].

SNP typing can be complementary to whole genome sequencing (WGS) when applied to TB and other pathogens [9, 12, 28–30]. Whole genome high throughput sequencing is probably the ultimate technique for microbial genotyping but even sequencing a proportion of isolates evolving in a clonal population structure such as MTB and subsequent bioinformatic analysis [31] will reveal SNPs uniquely associated with specific genotypes and will contribute to the optimization and interpretation of SNP screening assays.

Here, we report a strategy that we have developed to analyze, visualize and interpret SNP-based data generated by MLPA while minimizing the required user input. We assess the degree to which the interpretation of the results such as resistance profile and MTB lineage can be confidently assigned.

## Results

**Figure 1:**
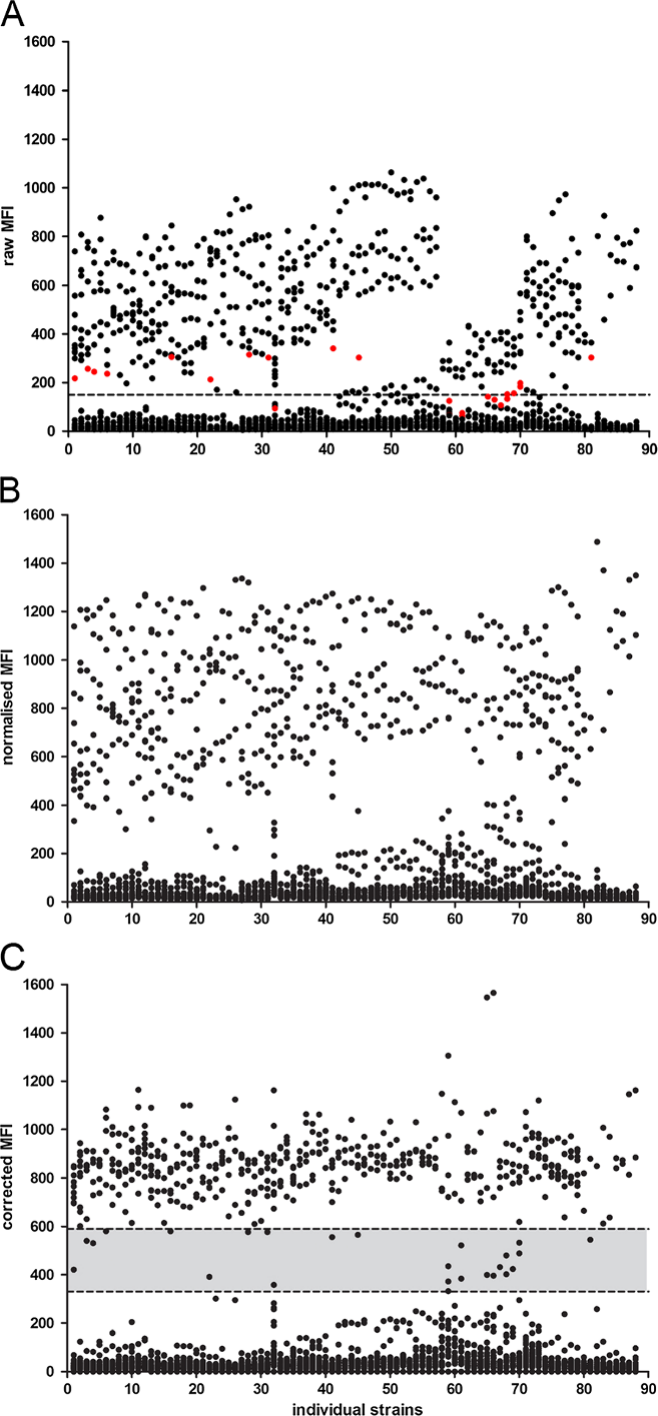
**Stepwise approach of the data analysis method.** Dot blots illustrate MFI values for 43 genetic markers targeted in 88 clinical isolates and laboratory strains [10] obtained from **(A)** the MAGPIX csv file, **(B)** after normalization and **(C)** after normalization and correction. **(A)** Raw MFI values obtained for every targeted marker per strain. The dashed line indicates the threshold of MFI 150 which was initially chosen for the classification of targeted makers. Red dots show the MFI values obtained which are located in the intermediate range after normalization and correction in panel **C**. **(B)** MFI values after intra-strain normalization of raw MFI values. **(C)** MFI signals after normalization and inter-strain correction using marker-specific correction factors. The grey area defines the intermediate range calculated as the area between one and two standard deviations from the average MFI_NORM_ = 860.

**Figure 2:**
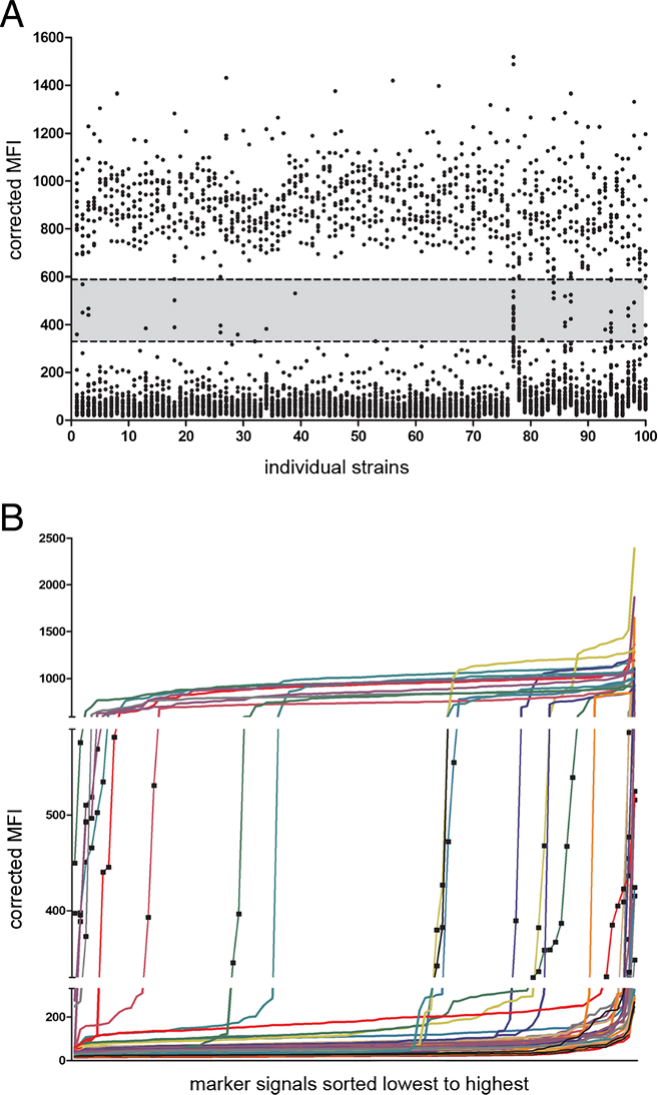
**Visualization of data generated from 100 Georgian isolates after data normalization and data correction. (A)** Dot plot showing normalized and corrected MFI values (black dots) per isolate for all 4300 markers targeted in the 100 Georgian isolates. The grey area highlights 62 (1.4%) unclassifiable markers of which 24 are drug resistance markers. Markers located above this area are classified as positive (971, 22.6%) and below as negative (3267, 76%). **(B)** Same data as shown in **(A)** but only the intermediate values are shown and visualized per marker. Each line shows the distribution of normalized and corrected MFI values, sorted from lowest o highest, (black squares), for one marker (individual colors).

The principle of the new data analysis method and the calculation of the correction factors is described in detail in the Methods section and in Figure 1. The results obtained with the new data analysis method from DNA of cultured isolates from individual patient samples collected at the National TB Reference Laboratory in Tbilisi, Georgia is illustrated in Figure 2.

After MLPA data normalization, correction and automated calling, 4238/4300 (98.6%) of the interrogated markers were classified as positive or negative (Figure 2). In total 62/4300 (1.4%) markers were classified as intermediate. The 62 intermediate calls were not constraint to specific strains or specific targeted markers, which would indicate an underlying structural error in the analysis (see Additional file 1). In 11 of the 26 strains with intermediate calls these calls were confined to drug resistance associated markers. The remaining 15 isolates required expert interpretation for assignment to an MTB lineage. For 74 of the 100 analyzed isolates a full MLPA profile was obtained without review by an expert.

**Figure 3:**
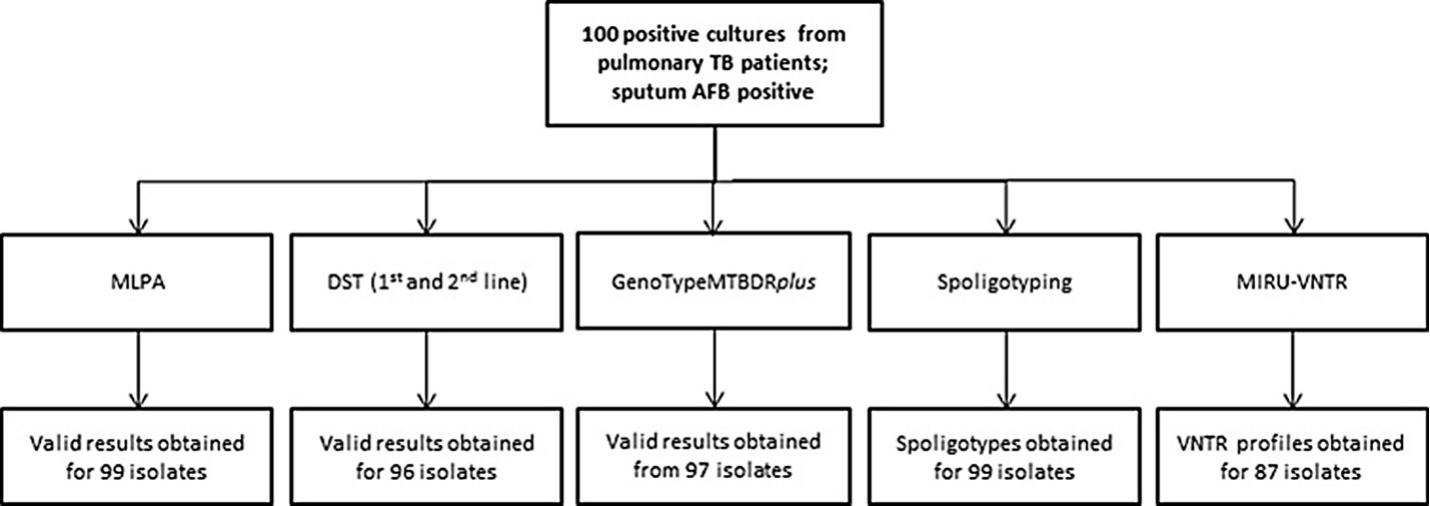
**Results obtained of the 100 isolates by various methods.** Samples were taken from 100 individual patients, all diagnosed with pulmonary TB and producing AFB positive sputum smear; MLPA, DST, GenoTypeMTBDR*plus,* spoligotyping and MIRU-VNTR was performed on all 100 isolates. DST for first line drugs was performed on all 100 isolates whereas DST for second line drugs was performed on drug-resistant TB isolates only. DST results were not available for four isolates due to contamination of the respective cultures; No information was obtained from three isolates tested by GenoTypeMTBDR*plus*; Spoligotypes were not obtained for one isolate. Unknown spoligotypes were obtained for 17 isolates and the spoligotypes of five isolates were not reported in the SITVITWEB database. MIRU-VNTR types were not obtained for 13 isolates. Multiple copy numbers in one or more loci were revealed in two isolates; Genotypic information of 45 markers screened per isolate was obtained for all 100 isolates by MLPA. For 85 isolates, lineage types could be assigned on the basis of consistent lineage marker profiles and in 14 isolates after expert review. For one isolate the lineage type profile was not interpretable. For 46 isolates molecular drug resistance was identified by MLPA of which 11 isolates had intermediate signals for at least one drug resistance conferring marker.

The results obtained from the MLPA and from the reference methods are summarized in Figure 3 and in Additional file 2. The MLPA produced interpretable profiles for 99 of the 100 isolates tested using the new data analysis method (Figure 3). The MLPA genotypes were called without user interpretation for 74 isolates, after expert review for a further 25 isolates and one isolate produced a profile that was uninterpretable.

To evaluate the accuracy of this calling strategy, all MLPA results were compared to results obtained from other methods. We used spoligotypes to validate the MLPA lineage types and the GenotypeMTBDR*plus* results as reference standard to validate the MLPA data obtained for the markers conferring resistance to first line drugs; DST was used as reference standard to validate the MLPA data obtained for the markers conferring resistance to second line drugs. In addition we have assessed the association between drug resistance and MLPA lineage (see Additional file 3) and MLVA clustering among all 100 isolates (see Additional file 4).

### Genotypic lineage identification

The genotypes identified by MLPA and spoligotyping are compared in Table 1. The MTB4 lineage was the dominant genotype by MLPA (64/99) of which 16 MTB4 isolates were further classified as Latin American-Mediterranean (LAM) and eight as Haarlem. Beijing was the second most commonly identified lineage (35/99), within which the MLPA identified four sublineages (Table 1). These results were fully supported by spoligotyping for all but five isolates (Table 1).

**Table 1:** **Comparison of MLPA lineage types and spoligotypes**

	Lineage type by spoligotyping																			
Lineage type by MLPA	Beijing	H1	H3	LAM1	LAM5	LAM9	T5-RUS1 (LAM) ^a^	T5-RUS1	MANU2	T1	T1-RUS2	T2	T3	T4	T5	Ural-1 ^b^	Unknown	Unknown (LAM) ^a^	Not reported before	Not-interpretable
Beijing K1 (n = 28)	28																			
Beijing V+/CHIN + (n = 5)	5																			
Beijing SA-/CHIN- (n = 1)	1																			
Beijing V- (n = 1)																	1			
MTB4 other than LAM or Haarlem (n = 40)			**1**						1	8	1		1	1	1	9	13		4	
LAM (n = 16)				3	1	1	2	**3**				**1**					3	1	1	
Haarlem (n = 8)		2	6																	
Non-interpretable (n = 1)																				1
Total (100 isolates)	34	2	7	3	1	1	2	3	1	8	1	1	1	1	1	9	17	1	5	1

Lineage types were obtained from spoligotyping using the SITVITWEB database. a, isolates were identified as T5-RUS1 or Unknown according to SITVITWEB but LAM according to their spoligotype identification type [41]; b, isolates were identified as Ural-1 on the basis of their SIT according to [42]; Numbers in bold indicate disagreement between MLPA and spoligotyping.

MIRU-VNTR typing data was obtained for 87 samples of which two isolates (C39 and C67) showed multiple copy numbers in seven loci (see Additional file 2). Cluster analysis of the MLVA types revealed 33 isolates with non-unique MLVA types, forming 10 clusters (see Additional file 4).

### Drug resistance

Results obtained from MLPA were compared to GenoTypeMTBDR*plus* (Table 2) and phenotypic DST results (see Additional file 5).

**Table 2:** **Correlation between drug resistance identified by MLPA and GenoTypeMTBDR** ***plus***

	Drug resistance by GenoTypeMTBDR***plus***									
Drug resistance by MLPA	rpoBWT	rpoBMUT3	rpoBMUT other ^a^	katGWT	katGMUT1	katGMUT other ^b^	inhAWT	inhAMUT1	inhAMUT1 and katGMUT1	No result
no rpoB mutation	76	0	3							2
intermediate rpoB	6	1	0							1
rpoB-S531L (rpoBMUT3)	0	11	0							0
rpoB-S522L	1	0	0							0
no katG mutation				74	0	0				1
katG-S315T (katGMUT1)				0	14	1				1
intermediate katG				1	0	0				0
no inhA mutation							64	0	0	2
inhA-15 C/T (inhAMUT1)							6	2	0	0
inhAMUT1 and katGMUT1							0	0	5	1
intermediate inhA							5	0	0	0
Total isolates (GenoTypeMTBDR*plus*)	82	12	3	75	14	1	75	2	5	3

Mutations obtained by MLPA are indicated for the respective genetic marker investigated with the GenoTypeMTBDR*plus* assay; isolates for which no results were obtained by GenoTypeMTBDR*plus* MLPA results for all genetic markers are indicated; an intermediate result indicates the presence of an intermediate signal and thus no presence or absence for the respective genetic marker; a = two isolates did not show a positive band with the rpoBWT8 probe but no mutation probe was positive; one isolate did not show a positive band for the rpoBWT3 and rpoBWT4. b = one isolate did not show a positive band with the katGWT1 probe but no mutation probe was identified.

Of the 700 loci screened for rifampicin and isoniazid resistance (*kat*G codon 315, *inh*A promoter region -15, *rpo*B codons 176, 522, 526G and 526 T and 531) by MLPA in the 100 isolates, 500 could be compared to data obtained by the GenoTypeMTBDR*plus* assay (loci *kat*G codon 315, *inh*A promoter region -15, *rpo*B codons 526G and 526 T and 531, the *rpoB* codons 176 and 522 could not be compared as they are only targeted by the MLPA assay. Results obtained by MLPA from 463 of the 500 (92.6%) loci compared were directly supported by the GenoTypeMTBDR*plus* assay (Table 2). One isolate was identified as isoniazid resistant based on the loss of the katGWT1 probe by the GenoTypeMTBDR*plus* assay (Table 2) and no mutation was identified by MLPA suggesting the presence of a less common mutation in *katG*. For the remaining 37 loci no results were obtained by the GenoTypeMTBDR*plus* assay for three isolates (15 loci) and for 14 of the remaining 22 loci intermediate values were obtained by MLPA. For the remaining eight (1.6%) loci there was a disagreement between the MLPA and the GenoTypeMTBDR*plus* assay.

The *rpoB*-S531L mutation was the only identified rifampicin resistance conferring mutation within the MDR-TB isolates (Table 2) both by MLPA and GenoTypeMTBDR*plus*. For three MDR isolates no mutation in *rpoB* was identified by GenoTypeMTBDR*plus* or MLPA but the absence of rpoBWT probes shown by the GenoTypeMTBDR*plus* suggests the presence of a less common mutation in *rpoB* (Table 2).

Current probes included in the MLPA for second line drug testing, target mutations conferring resistance to the injectable drugs amikacin, kanamycin and capreomycin (rrs1401), capreomycin (rrs1402) or fluoroquinolones (gyrA90 and gyrA94). MLPA identified resistance to amikacin, kanamycin and capreomycin in two of six MDR-TB isolates resistant to kanamycin and capreomycin by DST (see Additional file 5). Two isolates showed resistance to ofloxacin by DST, but no fluoroquinolone resistance was detected by MLPA in any of the 100 isolates.

DST identified 16 isolates with ethambutol resistance. A mutation in the 306 codon of the *embB* gene conferring resistance to ethambutol was confidently detected by MLPA in only four isolates all of which were also phenotypically resistant (see Additional file 5). Additionally MLPA detected an intermediate value for ethambutol resistance in two isolates one of which was determined ethambutol resistant by DST (see Additional file 5). MLPA identified a mutation in the codon 43 of the *rpsL* gene conferring resistance to streptomycin in 11 of 52 phenotypically streptomycin resistant isolates (25%) and in one the 44 (2.2%) phenotypically sensitive isolates. In one of the 52 isolates that were phenotypically streptomycin resistant an intermediate signal for the rpsl-43 marker was obtained.

## Discussion

We believe that an easily interpretable, robust, high-throughput multiplex genotyping method such as MLPA can support infectious disease monitoring and control. Critically unlike many typing methods SNP-based genotyping is directly compatible with data generated by WGS/next generation sequencing (NGS). Synergy between SNP-based surveillance and SNP discovery could be facilitated by cost-effective high throughput assays. Moreover, routine screening of clinical isolates with WGS is currently not feasible in most high prevalence MDR-TB settings, while a robust and dedicated method like MLPA is more practical to implement. Also, the read out platform used here, the Luminex technology is relatively robust and suitable for other assays notably the newly developed TB-SPRINT method [32].

As a result of the clonal population structure of the MTB complex, genotypic information such as SNPs and large sequence polymorphisms that are not under strong selective pressure can be used to identify specific genotypic lineages and are unique and mutually exclusive. Indeed, many possible MLPA profiles are invalid, as MTB is clonal, a pure MTB strain will contain all and only genetic markers from a single lineage [8, 9]. This allows an internal quality check, unlike classical typing methods for example IS6110 RFLP typing, or VNTR typing and to some extent spoligotyping [33–35] where virtually all profiles are theoretically possible.

Here we have analysed a panel of 100 isolates from patients diagnosed with pulmonary TB and with positive sputum smear microscopy collected in 2011 at the NCTBLD in Tbilisi, Georgia and used the data generated to evaluate a novel data analysis strategy. From the 100 isolates analyzed, 74 were confidently assigned to an MTBC lineage by our algorithm without expert interpretation. After expert review of the remaining 26 MLPA profiles only a single profile remained uninterpretable. The results of these 99 profiles were compared to reference methods.

Previously we called positives on the basis of a threshold MFI (equal or higher than 150) with no intermediate signals [10]. Analyzing the data presented here using the threshold method would have resulted in 951 of the 4300 characteristics being called positive and 3349 negative. Using our new data analysis method 971 characteristics were called positive and 3267 called negative, 62 MFI signals were classified as intermediate. The 62 intermediate calls were distributed amongst 26 isolates, and 23 of the 62 (37%) were markers related to drug resistance distributed amongst 11 isolates. Of these 62 intermediate calls 13 would have been called positive by the original analysis method (four correctly, seven incorrectly and two unknown due to the lack of a reference standard) and 49 negative (21 correctly, 10 incorrectly, 18 unknown). With respect to lineage identification the threshold method would have resulted in 22 inconsistent profiles in contrast to 14 profiles flagged for expert review (of which 13 could be resolved) with the new data analysis method.

Importantly, as we normalize with pre-established marker correction factors a single profile can be interpreted and no minimum sample size is required to validate the calls in contrast to other approaches [36]. Those profiles containing of intermediate signals or those with a profile inconsistent with established lineage identification are flagged and need to be inspected by an expert. This approach means that samples with poor DNA quality, experimental error or experimental variability are identified and not spuriously classified automatically. Figure 2B shows that for each marker, when the MFI values are ordered from lowest to highest, for the 100 isolates analyzed each marker ideally follows a sigmoid curve with only very few values in the intermediate range – these are the intermediate calls.

In the Georgian region a diverse phylogeny of MTB has been previously observed [25, 26, 37]. Genotyping by MIRU-VNTR, spoligotyping and MLPA of 100 randomly sampled isolates from this region here supports this diversity as well as allowing us to validate our assay by comparing the genotypes obtained by each method. Spoligotypes were obtained for 99 of the 100 isolates of which 17 were unknown (ambiguous) in the SITVITWEB database and five were not previously reported (Table 1). For the 77 isolates for which an MLPA profile and known spoligotype was available 72 (93.5%) matched. The one isolate with no MLPA profile available was “direct repeats” negative by spoligotyping. The 22 isolates with unknown or not previously reported spoligotypes were all classified to a specific lineage type by MLPA (Table 1) these classifications were supported by MIRU-VNTR (see Additional file 2).

Genetic markers under strong selective pressure, most notably antimicrobial resistance associated mutations, may occur in multiple lineages [19]. Some lineages have been suggested to be more associated with (multi-) drug resistance and therefore identification of some lineages may be particularly important for public health (reviewed in [1]). Data collected between 2003 and 2005 in the WHO European Region and elsewhere shows that majority of MDR-TB isolates belonged to the Beijing lineage [2]. An association of the Beijing lineage with multi-drug resistance has previously been also observed in Georgia [25–27] and in the study presented here a member of the Beijing lineage was 7.4 × (37% versus 5%) more likely to also have an MDR profile than members from other lineages. In order to confirm these findings testing larger numbers of epidemiologically more representative sample sets needs to be undertaken. Methods such as MLPA are suitable to undertake these studies; for example even in this limited initial study the Beijing clone K1, MLVA type 94–937 was for the first time identified in Georgia for four isolates two of which were MDR (see Additional file 4).

Intermediate calls might indicate the presence of sensitive and resistant alleles. One isolate that was MDR-TB by GenotypeMTBDR*plus* had an intermediate signal for rpoB-531 and embB306 by MLPA after data analysis, possibly indicating heteroresistance or mixed infection. Further evaluation of the data analysis method is required to prove the ability of the current MLPA to detect heteroresistance. The acquisition of resistance during MDR-TB therapy has also been documented, as was detected by MLPA, in South Africa and shown to affect the performance of molecular assays that detect resistance to second line drugs [38]. Two studies report the occurrence of drug resistance amplification and patients infected with more than one genotype in Georgia [27, 39]. Resistance amplification and re-infection with MDR-TB isolates could be partially accountable for the high failure rates in especially new TB patients in Georgia [21].

At present a limitation of the MLPA assay is that it is only suitable for use on cultured isolates but developments in real time assays appear to provide the possibility to perform an MLPA assay in a closed tube format [40] which could dramatically simplify implementation.

## Conclusions

At the NCTBLD in Tbilisi, Georgia, no method for extensive genotyping or strain identification was implemented prior to this work. Therefore, lineage identification and phylogenetic analysis need to be performed outside of the NCTBLD. As a result of this study, the development of a transparent analysis algorithm, and associated staff training the MLPA method is now able to be performed on site and a report of the first year’s data is in preparation. Even when performed locally on cultures, rather than directly on sputum, combined lineage identification and screening for drug resistance can provide much needed insight into the genotypic background of circulating drug resistant strains in a more timely manner.

## Methods

### Bacterial/DNA samples

Positive cultures from 100 individual patients referred to the NRL, Tbilisi, Georgia were collected. Isolates were randomly selected from patient samples between January and April 2011 with a positive acid-fast bacilli (AFB) microscopy result and diagnosed with pulmonary TB; 28 were from patients previously treated for TB. Ethical approval was not required for this study as no patient information was used and the results of the analysis of the bacterial cultures could not be linked back to individual patients.

Within the routine workflow for TB patient sample testing at the NRL the following data was collected: Sputum samples were directly inoculated onto LJ –based solid medium and BACTEC MGIT 960 system (BD, Sparks, MD, USA) drug susceptibility testing (DST) against first-line drugs for isoniazid and rifampicin was performed using the absolute proportion method on Löwenstein-Jensen (LJ) medium or the BACTEC MGIT 960 system [23] and molecular drug resistance testing for isoniazid and rifampicin using the Genotype MTBDR*plus* assay (Hain Lifescience, Nehren, Germany) [22]. Routine Genotype MTBDR*plus* assay results were obtained directly from DNA extracted from sputum only if the sputum smear was positive and the sample was no older than four days, otherwise MTBDR*plus* was performed on subsequent positive cultures. All isolates identified as drug-resistant-TB were additionally subjected to second line DST (proportion method on LJ medium) [23]. DST against the second-line drugs ethionamide (Eto), ofloxacin (Ofx), para-amino-salicylic acid (PAS), capreomycin (Cm) and kanamycin (Km) was performed using the proportion method on LJ medium as previously described [23].

DNA extracted for routine GenoTypeMTBDR*plus* testing was also used for MLPA analysis. In brief, 1 ml bacterial culture was taken from a respective BACTEC MGIT culture. Bacterial cells were pelleted and resuspended in 100 μl molecular grade water. DNA was released by thermolysis at 95° Celsius for 30 min and sonication for 15 min. Cell debris was pelleted and the supernatant containing the DNA was stored at -20° Celsius. MLPA analysis was performed, as explained below, without any knowledge of the routine test results at KIT Biomedical Research (Royal Tropical Institute), Amsterdam.

### MLPA

The bead-based MLPA was performed as previously described with an identical 43-plex probe mix [10] on a MAGPIX® device (Luminex Corp., Austin, Texas, USA). In brief, 13 drug resistance markers and 30 phylogenetic informative markers were targeted in the mycobacterial genome and two controls LumH and LumD were run with every sample. For quality control of the bead-based MLPA four control samples are run with every MLPA experiment [10]: assay control, no template control, contamination control and MAGPIX control. Molecular markers associated with resistance to streptomycin (S), isoniazid (I), rifampicin (R), and ethambutol (E) are included targeting respectively the wildtype allele in the *rpsl-*43 locus, *katG* (AGC 315 ACC) and *inhA* (-15C/T), *rpoB* (GTC 176 TTC), *rpoB* (TCG 522 TTG), *rpoB* (CAC 526 GAC), *rpoB* (CAC 526 TAC), *rpoB* (TCG 531 TTG) and the wildtype allele in the *embB* 306 locus. Mutations associated with molecular resistance to the injectable drugs amikacin (Am), kanamycin (Km) and capreomycin (Cm) are also targeted by inclusion of probes for the wildtype allele in *rrs* (1401) (Am, Km, Cm) and *rrs* (1402 C/T) (Cm). The fluoroquinolone (FLQ) resistance associated mutations *gyrA* (GCG 90 GTG) and *gyrA* (GAC 94 GGC) were also targeted.

The assignment to lineages within the MTB complex was based on the previously published algorithm [10]) and comprehensive MTB complex phylogeny [8–10]. A slightly modified version of the algorithm proposed in [10] was used for the identification of MTB lineages and sublineages (see Additional file 6). The markers RD-seal and RD1-mic were added for the identification of *Mycobacterium microti* or *Mycobacterium pinnepedii*, respectively and the algorithm for identification of non-tuberculous mycobacteria was removed. Raw MFI signals obtained for all samples from the MAGPIX csv-file are compiled in Additional file 7.

### MLPA data analysis

The csv-file produced after every experiment by the Luminex xPonent 4.2 software was imported into an Excel worksheet (Microsoft, Redmond, USA). First an intra-sample normalization (1. below) on the raw Median Fluorescence Intensity (MFI) signals was performed to allow inter-sample comparisons. This was followed by the application of marker-specific correction factors (2. below), which simplifies interpretation and visualization of the results. The MFI signals from the controls LumD and LumH were excluded from the data normalization process.

1. Calculation of MFI_NORM._

On the basis of the currently used MLPA probes and an optimally performing MLPA assay, at least five markers will give a true positive signal in every MTB isolate with a good quality DNA independent of the drug resistance profile or lineage type. The normalized MFI value (MFI_NORM_) represents the raw MFI signal (Figure 1A) of each probe per sample of a run multiplied by an arbitrary factor (5000) and divided by the sum of the first five highest MFI signals per isolate (Figure 1B). The value of 5000 as numerator was chosen to facilitate visualization of the data only. It does not qualitatively influence the data results.

2. Calculation of MFI_CORR_

To ease interpretation and allow a single cut-off to be applied for each marker all normalized MFI values were multiplied with previously calculated marker-specific correction factors.

#### Calculation of marker-specific correction factors

For the calculation of marker-specific correction factors previously published data from 88 well characterized laboratory strains and clinical isolates [10] were normalized as in (i) above. Then the average MFI_NORM_ was calculated for each marker (e.g. inhA-15) using only the true positive signals of the marker. The overall average MFI_NORM_ was calculated subsequently by adding all marker-specific MFI_NORM_ averages and dividing by the number of all markers. The average MFI_NORM_ was 850. We also calculated +1 and -1 standard deviation (SD) of the average MFI_NORM_ (average MFI_NORM_ = 850, 1^st^ SD +/ - 260). The area of low mathematical confidence calls was determined to be between -1 and -2 SD from the average of all positives (MFI_CORR_ 590 – 330) (Figure 1C, grey area). Marker-specific correction factors were calculated by dividing the overall average MFI_NORM_ (value of 850) with the marker-specific average MFI_NORM_.

All MFI_NORM_ values of a new dataset, here the 100 isolates, were multiplied with the calculated standard marker-specific correction factors calculated above resulting in the corrected MFI (MFI_CORR_) (Figure 2A-B). Visualization of the raw data with respect to isolate (Figure 2A) and marker (Figure 2B) is shown. Calling of the results: For each isolate analyzed the genotype was called if no intermediate genotypic markers were present or flagged for final interpretation by an expert if the MFI_CORR_ of one or more genotypic markers was in the grey area (intermediate). Thus genotype profiles were called without user interpretation, resolved by an expert or identified as un-interpretable by an expert.

### Spoligotyping

Spoligotyping was performed at the University Paris-Sud on a Luminex 200® and/or on the MAGPIX® platform on all 100 samples using 43 spacer-spoligotyping [34] on an aliquot of the same DNA sample used for the MLPA analysis. Spoligo International Type (SIT) numbers and lineages were assigned according to the SITVITWEB database [33] and adapted according to [41, 42]. Octal and binary codes reported in the SITVITWEB database that do not provide lineage information are referred to as ‘unknown’. Octal and binary codes that are not identified in the SITVITWEB database are referred to as ‘not reported before’.

### MIRU-VNTR typing

Standard VNTR typing using 24 loci was performed as previously described by Supply *et al.* [43] and optimized by the RIVM [44] at the RIVM in Bilthoven. Identification of MLVA 15–9 codes was carried out by using the MIRU-VNTR*plus* database [45]. A cluster was defined as a minimum of two isolates with identical MIRU-VNTR patterns.

### Statistics

A One-tailed Fisher’s exact test was used to statistically assess if the MDR phenotype was equally distributed amongst the genotypes Beijing and non-Beijing. The P-value was calculated using a two-by-two table with Beijing and MDR (13 isolates), Beijing and non-MDR (21 isolates), non-Beijing and MDR (3 isolates), non-Beijing and non-MDR (58 isolates).

## Electronic supplementary material

Additional file 1: Table S1: Intermediate calls stratified per individual marker. (XLSX 12 KB)

Additional file 2: Table S2: Summary of data obtained for 100 isolates. (XLSX 408 KB)

Additional file 3: Table S3: DST results for 100 Georgian isolates stratified according to the MLPA lineage type. (XLSX 9 KB)

Additional file 4: Table S4: MLVA clusters identified in 100 Georgian isolates. (XLSX 12 KB)

Additional file 5: Table S5: Correlation between drug resistance identified by MLPA and DST. (XLSX 10 KB)

Additional file 6: **Algorithm applied to all isolates analyzed for lineage identification of** ***Mycobacterium tuberculosis*** **complex.** MLPA markers are framed and final MTB complex lineages or sublineages are shown in bold. The species identification of a sample starts with the MTB complex 16SrRNA marker. As an example the call for the Beijing lineage K1 is highlighted with bold arrows. The following markers are present or absent in an isolate belonging to the Beijing K1 lineage: MTBC 16S rRNA (present), TbD1 (present), RD750 (RD sequence present), pks15/1–7 (absent), RD105 (RD sequence deleted), fbpB-238 (present), mutT2-58 (present), acs-1551 (absent), RD131 (RD sequence deleted). MTBC, MTB complex. EAI, East African Indian; CAS, Central Asian; LAM, Latin American Mediterranean; Updated version of [10]. (JPEG 632 KB)

Additional file 7: Table S7: Raw MFI data of the MLPA assay. (XLSX 38 KB)

## Abbreviations

TB: Tuberculosis
MTBC: *Mycobacterium tuberculosis* complex
SNP: single nucleotide polymorphism
MDR: Multidrug resistance
DNA: Deoxyribonucleic acid
MLPA: Multiplex ligation-dependent probe amplification
DST: Drug susceptibility testing
MIRU-VNTR: Multiple interspersed receptive units – variable number of tandem repeats
MLVA type: Multiple locus variable number tandem repeat analysis type
SIT: Spoligo International Type
MFI: Median fluorescence intensity
WGS: Whole genome sequencing
S: Streptomycin, anti-TB drug
I: Isoniazid, anti-TB drug
R: Rifampicin, anti-TB drug
E: Ethambutol, anti-TB drug
Km: Kanamycin, anti-TB drug
Am: Amikacin, anti-TB drug
Cm: Capreomycin, anti-TB drug
Eto: Ethionamide, anti-TB drug.
