# Supplementary figures and images for: Optimizing multiplex SNP-based data analysis for genotyping of Mycobacterium tuberculosis isolates
Source: BMC Genomics. 2014 Jul 7;15(1):572. doi: 10.1186/1471-2164-15-572 (PMC4117977; doi:10.1186/1471-2164-15-572)

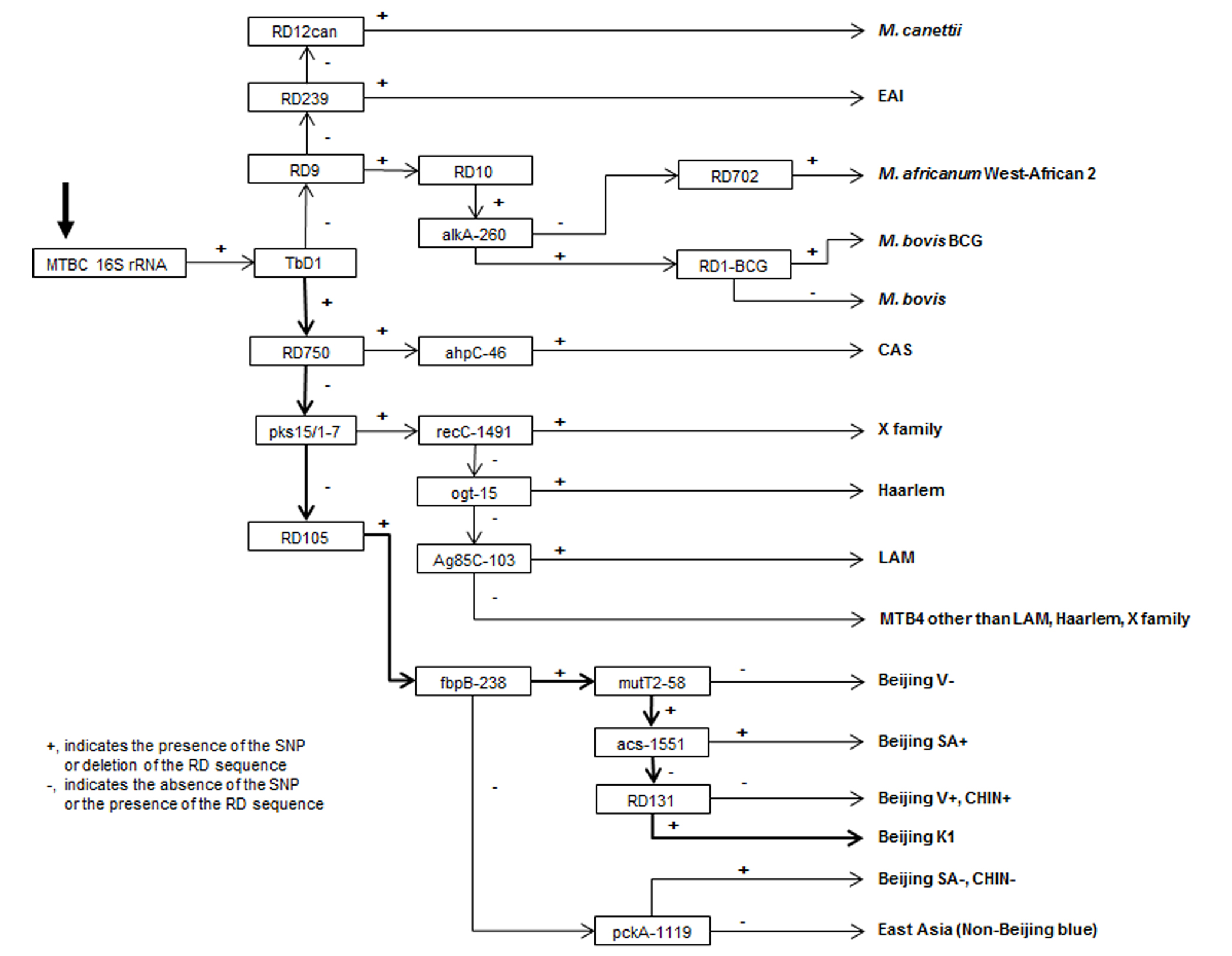

Supplement: Supplementary file 6 — Additional file 6: Algorithm applied to all isolates analyzed for lineage identification of Mycobacterium tuberculosis complex. MLPA markers are framed and final MTB complex lineages or sublineages are shown in bold. The species identification of a sample starts with the MTB complex 16SrRNA marker. As an example the call for the Beijing lineage K1 is highlighted with bold arrows. The following markers are present or absent in an isolate belonging to the Beijing K1 lineage: MTBC 16S rRNA (present), TbD1 (present), RD750 (RD sequence present), pks15/1–7 (absent), RD105 (RD sequence deleted), fbpB-238 (present), mutT2-58 (present), acs-1551 (absent), RD131 (RD sequence deleted). MTBC, MTB complex. EAI, East African Indian; CAS, Central Asian; LAM, Latin American Mediterranean; Updated version of [10]. (JPEG 632 KB) [file 12864_2014_6299_MOESM6_ESM.jpeg]
